# Improved Synthesis of Cu_2_O NPs and Ascorbic Acid-Modified Derivatives for Adsorption of Brilliant Cresyl Blue: Surface and Reusability Studies

**DOI:** 10.3390/ma17102358

**Published:** 2024-05-15

**Authors:** Saad Zeghdi, Salah Eddine Laouini, Hamdi Ali Mohammed, Abderrhmane Bouafia, Mohammed Laid Tedjani, Mahmood M. S. Abdullah, Tomasz Trzepieciński

**Affiliations:** 1 Laboratory of Recovery and Promotion of Saharan Resources, Department of Chemistry, Faculty of Mathematics and Material Sciences, University Kasdi Merbah, Ouargla 30000, Algeria; saad.zeghdi@gmail.com; 2 Department of Process Engineering, Faculty of Technology, University of El Oued, El-Oued 39000, Algeria; hamdimohammed116@gmail.com (H.A.M.); abdelrahmanebouafia@gmail.com (A.B.); medlaidtedjani@gmail.com (M.L.T.); 3 Laboratory of Biotechnology Biomaterial and Condensed Matter, Faculty of Technology, University of El Oued, El-Oued 39000, Algeria; 4 Department of Chemistry, College of Science, King Saud University, P.O. Box 2455, Riyadh 11451, Saudi Arabia; maltaiar@ksu.edu.sa; 5 Department of Manufacturing Processes and Production Engineering, Rzeszow University of Technology, Al. Powstańców Warszawy 8, 35-959 Rzeszów, Poland; tomtrz@prz.edu.pl

**Keywords:** Cu_2_O nanoparticles, ascorbic acid, derivative photocatalysis, enhanced, dyes degradation

## Abstract

This study addresses the critical need for efficient and recyclable photocatalysts for water treatment applications by presenting a novel approach for the synthesis and characterization of copper (I) oxide (Cu_2_O) nanoparticles modified with ascorbic acid (Cu_2_O/AA). The motivation for this research stems from the increasing concern about environmental pollution caused by organic pollutants, such as Brilliant Cresyl Blue (BCB), and the necessity for sustainable solutions to mitigate this issue. Through comprehensive characterization techniques including Ultraviolet–Visible spectroscopy (UV-Vis), Fourier Transform Infrared spectroscopy (FTIR), X-ray Diffraction (XRD), Scanning Electron Microscopy (SEM), zeta potential measurements, and Brunauer–Emmett–Teller (BET) analysis, we demonstrate a significant modification to the electronic structure, enhancing the photocatalytic activity of Cu_2_O/AA. BET analysis revealed a mesoporous structure with a specific surface area of 2.7247 m^2^/g for Cu_2_O/AA, further emphasizing its potential for enhanced catalytic performance. The photocatalytic degradation studies showcased remarkable efficiency improvements, with degradation coefficients of 30.8% and 73.12% for Cu_2_O NPs and Cu_2_O/AA NC, respectively, within a 120 min timeframe. Additionally, recyclability experiments indicated sustained efficiency over five consecutive cycles, with both catalysts retaining crystalline integrity. These findings underscore the promising potential of Cu_2_O/AA nanoparticles as highly efficient and recyclable photocatalysts for the degradation of organic pollutants, offering superior performance compared to pure Cu_2_O NPs and addressing the pressing need for sustainable water treatment solutions.

## 1. Introduction

Industrialization has brought about immense progress, but it also carries a significant environmental cost, particularly in the form of water pollution [1,2]. Various industries generate organic dyes that are toxic to aquatic life, posing a severe threat to fragile ecosystems. Among the various contaminants, synthetic dyes represent a considerable portion, with Brilliant Cresyl Blue (BCB) being one of the commonly used dyes in the textile industries [3,4]. Due to its complex aromatic structure and persistence, BCB poses a significant threat to aquatic ecosystems and human health, necessitating effective remediation strategies [5]. Researchers have been actively seeking effective methods to remove these harmful dyes from wastewater, and one of the most promising approaches is the use of photocatalysts. Photocatalytic degradation, which employs semiconductor photocatalysts, has emerged as a cost-effective and environmentally friendly solution for breaking down these dyes [6,7]. By harnessing the power of light and catalytic materials, this method offers a sustainable and efficient way to mitigate the detrimental effects of industrial dye effluents on water bodies, paving the way for a cleaner and more sustainable future [8,9].

Semiconductor photocatalysts, such as copper oxide nanoparticles (Cu_2_O NPs), are capable of generating reactive oxygen species (ROS) upon exposure to light. This process begins with the absorption of photons by the photocatalyst, which is sufficient to excite electrons to a higher energy level, thereby creating electron-hole pairs in the semiconductor’s conduction and valence bands [10,11]. These charge carriers can then interact with adsorbed water and oxygen molecules on the photocatalyst’s surface, initiating redox reactions [12].

The ROS produced through these redox reactions, including hydroxyl radicals ${OH}^{\cdot }$ and superoxide ions ${O_{2}^{\cdot }}^{-}$, play a crucial role in the degradation of organic pollutants [13]. Les ${OH}^{\cdot }$ radicals are particularly reactive and can oxidize a wide range of organic compounds, leading to their decomposition into smaller, less toxic molecules [14]. This ability to decompose pollutants makes photocatalytic degradation a promising and effective method for water treatment.

Photocatalytic degradation has emerged as a promising approach for the removal of organic pollutants from water. This process harnesses the light-induced activity of semiconductor materials to generate reactive oxygen species (ROS), which effectively degrade organic molecules into harmless byproducts [15,16,17]. Among the various semiconductor materials investigated for photocatalytic applications, cuprous oxide nanoparticles (Cu_2_O NPs) have garnered significant attention due to their favorable bandgap energy, abundant availability, low cost, and environmentally friendly nature [18,19]. Cu_2_O NPs exhibit excellent photocatalytic properties, making them suitable candidates for the degradation of organic pollutants [20]. However, the practical application of Cu_2_O NPs in wastewater treatment is often hindered by challenges such as limited stability, rapid recombination of photogenerated charge carriers, and inefficient utilization of visible light [21,22].

To address these limitations and enhance the photocatalytic performance of Cu_2_O NPs, surface modification techniques have been explored to tailor their properties and improve their efficiency in dye degradation processes. Ascorbic acid (AA), a naturally occurring antioxidant and reducing agent, has emerged as a promising modifier for Cu_2_O NPs due to its ability to stabilize nanoparticles, inhibit electron–hole recombination, and enhance light absorption properties. The incorporation of ascorbic acid onto the surface of Cu_2_O NPs can potentially lead to synergistic effects, resulting in improved photocatalytic activity and stability [23]. Kader and coworkers investigated the efficacy of L-ascorbic acid adduct-conjugated ZnONPs in the degradation of Congo red, yielding promising results [24,25]. By functionalizing the nanoparticle surface with ascorbic acid, researchers can enhance charge separation efficiency, mitigate surface defects, and facilitate the generation of ROS, thereby augmenting the photocatalytic degradation capabilities of the nanoparticles [23,26].

The synthesis of nanoparticles through chemical processes stands as a cornerstone in the realm of nanomaterial fabrication, offering precise control over their size, morphology, and surface properties. Chemical methods provide a versatile platform for tailoring the characteristics of nanoparticles, allowing researchers to manipulate parameters such as precursor composition, reaction conditions, and stabilizing agents to achieve desired material properties [27,28]. In particular, the chemical synthesis of Cu_2_O NPs has garnered significant attention due to its simplicity, scalability, and potential for precise control over nanoparticle attributes [29,30]. By judiciously selecting reactants and optimizing reaction parameters, researchers can dictate the nucleation and growth kinetics, leading to the formation of Cu_2_O NPs with tailored sizes and morphologies. Moreover, chemical synthesis routes facilitate the incorporation of surface modifiers and functional groups, further enhancing the nanoparticles’ stability and reactivity [31,32]. Thus, leveraging chemical processes for the synthesis of Cu_2_O NPs holds immense promise for advancing various applications, including catalysis, sensing, and environmental remediation [33].

In this study, we present a novel approach by synthesizing and characterizing cuprous oxide (Cu_2_O) nanoparticles modified with ascorbic acid (Cu_2_O/AA) for enhanced photocatalytic degradation of Brilliant Cresyl Blue (BCB). Through chemical synthesis and detailed characterization techniques including UV-Vis spectroscopy, FTIR, XRD, SEM, zeta potential measurements, and Brunauer–Emmett–Teller (BET) analysis, this research elucidates the synergistic effects of nanoparticle composition and surface modification on catalytic performance. The primary application lies in advancing water treatment technologies by efficiently removing organic pollutants from aqueous environments. By uncovering the mechanisms of photocatalytic degradation and assessing practical applicability, this study aims to contribute to environmental remediation strategies with potential implications for wastewater treatment, catalysis, and sensing.

## 2. Method and Materials

### 2.1. Materials

Copper chloride (CuCl_2_, 99%), sodium hydroxide (NaOH), ascorbic acid (C_6_H_8_O_6_), Brilliant Cresyl Blue (BCB) dye were procured from Sigma-Aldrich, Darmstadt, Germany.

### 2.2. Synthesis of Cu_2_O NPs

The method of synthesizing Cu_2_O NPs was described in a previous research paper, with slight modifications implemented for this particular study [28]. In brief, a reducing agent, 1 M NaOH, was incrementally introduced into a solution containing 100 mL of 2 M copper chloride (CuCl_2_) while it was stirred for a duration of 5 min at 65 °C. Following this, the pH of the mixture was meticulously adjusted to 12.5 using a 2 M NaOH solution. Subsequently, the resulting solution was subjected to agitation for 2 h, leading to a discernible alteration in color to a dark violet blue. The subsequent isolation of the precipitate was accomplished through centrifugation at 3000 rpm for 5 min, accompanied by successive washings with distilled water (DW) to eliminate any impurities. The resultant precipitate underwent a drying process at 80 °C for approximately 15 h. A critical step in the synthesis protocol involved the annealing of the dried powder in an oven at 500 °C for 4 h, which played a pivotal role in both initiating and stabilizing the desired nanoparticle. This comprehensive procedure ensured the successful fabrication of Cu_2_O NPs.

### 2.3. Ascorbic Acid-Mediated Cu_2_O Nanoparticles

The obtained Cu_2_O NPs surfaces were modified with ascorbic acid according to previous study with slight modifications [29]. Initially, 0.5 g of Cu_2_O NPs was dispersed in 50 mL of distilled water and stirred at ambient temperature for 10 min. Subsequently, ultrasonic dispersion of the Cu_2_O NPs solution was conducted at 50 °C for 2 h. The pH of the solution was regulated to 6.5 by the addition of an ascorbic acid stock solution (0.1 g/mL). The reaction mixture was maintained under constant stirring at 70 °C for 2 h. Following the reaction, the resulting suspension was subjected to drying in a vacuum oven at 50 °C for 5 h, yielding ascorbic acid-stabilized Cu_2_O NPs (Cu_2_O/AA).

### 2.4. Characterization of Cu_2_O NPs and Cu_2_O/Ascorbic Acid NPs

The characterization of Cu_2_O NPs and Cu_2_O/ascorbic acid (AA) NPs was conducted using a range of analytical techniques. UV–visible spectrophotometry (SECOMAM, model 9600, Alès, France) was employed to analyze the optical properties of the synthesized NPs. X-ray diffraction (XRD) analysis (Proto Manufacturing Company’s Benchtop model, Maple Plain, MN, USA) was utilized to determine the crystallite size employing the Scherrer formula [34], where a prominent peak with the highest intensity was selected. Fourier transform infrared spectroscopy (FTIR) (Thermo Fisher Scientific, Nicolet iS5 model, Waltham, MA, USA) was employed to investigate the chemical composition and functional groups present on the surface of the NPs. Scanning electron microscopy (SEM) (TESCAN, VEGA3 model, Warrendale, PA, USA) provided insight into the morphology and size distribution of the NPs. Zeta potential measurements were carried out using the Entegris company’s Nicomp Nano Z3000 model (Billerica, MA, USA) with Dynamic Light Scattering (DLS) sizing capability to assess the surface charge and stability of the NPs. Additionally, the Brunauer-Emmett-Teller (BET) method (Model Nova 2000e, Quantachrome Instruments Limited, Boynton Beach, FL, USA) was employed to determine the specific surface area and pore size distribution of the synthesized NPs. These comprehensive characterization techniques facilitated a thorough understanding of the structural, optical, morphological, and surface properties of both Cu_2_O and Cu_2_O/AA NPs, crucial for evaluating their potential applications in various fields [35,36].

### 2.5. Photocatalytic Activity

The investigation assessed the photocatalytic capabilities of Cu_2_O NPs and Cu_2_O/ASA NPs in a solution containing Brilliant Cresyl Blue (BCB) dye. A solution was prepared, comprising 200 mL of BCB with a concentration of 50 parts per million (PPM). The impact of contact time was methodically evaluated at various intervals (5, 15, 30, 45, 60, 90, and 120 min) utilizing Cu_2_O NPs and Cu_2_O/ASA NPs at a concentration of 5 mg/5 mL. After the treatment, the nanoparticle samples were separated by centrifugation, and the UV-visible absorbance spectra of the BCB dye solution were analyzed using a spectrophotometer across the wavelength range of 200–800 nm. The degradation efficiency of BCB dye was quantified according to the provided equation [37].

(1) $$D\;(\%\;degradation)=\frac{A_{0}-A_{\left(t\right)}}{A_{0}}\times 100$$

where *D* represents the efficiency of degradation rate, while *A*_0_ and *A_t_* signify the initial absorbance and the absorbance at a given time point, respectively.

## 3. Results and Discussion

### 3.1. UV-Vis Analysis

**Figure 1:**
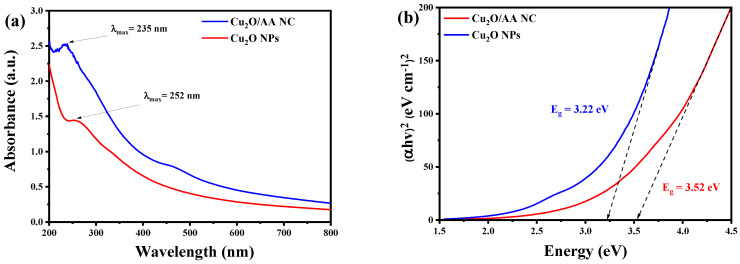
(**a**) UV-Vis spectra of Pure Cu_2_O and Cu_2_O/AA NPs; (**b**) band gap energy of Pure Cu_2_O and Cu_2_O/AA NPs. The arrows are did designed to determine the gap energy for each sample in the row X.

In Figure 1a, the UV-Vis absorption spectra of Cu_2_O NPs and Cu_2_O/AA NPs are presented. As seen, the Cu_2_O NPs exhibit a lower intensity absorbance at 252 nm. In contrast, the Cu_2_O/AA NPs display a higher intensity absorbance at a slightly shorter wavelength of 235 nm. The shift in wavelength suggests a change in the electronic structure or environment of Cu_2_O upon interaction with ascorbic acid, potentially leading to surface modification or electronic interactions between the two components. The increased absorbance intensity in the presence of ascorbic acid indicates enhanced light absorption or a change in the band structure.

To further elucidate the optical properties, the band gap energy for both of Cu_2_O and Cu_2_O/AA NPs was determined utilizing Tauc’s relation [38]. By plotting (hv)^2^ against energy (eV), the band gap energy was computed, yielding values of 3.52 eV and 3.22 eV, respectively, as shown in Figure 1b. These values provide insights into the energy required for electronic transitions within the materials. The lower band gap energy required by Cu_2_O/AA NPs compared to pure Cu_2_O suggests a modification in the electronic structure due to the presence of ascorbic acid. This alteration in band gap energy further corroborates the UV-Vis absorption results, indicating changes in the optical properties of Cu_2_O upon interaction with ascorbic acid.

### 3.2. FTIR Analysis

**Figure 2:**
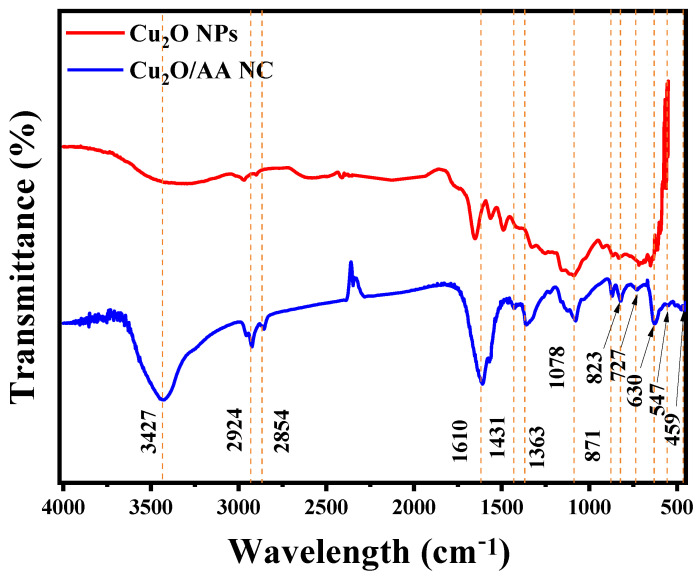
FTIR spectra of Cu_2_O and Cu_2_O/ascorbic acid.

Figure 2 illustrates the FTIR spectrum of the Cu_2_O/AA NPs, displaying distinctive peaks associated with the identified constituents. These peaks offer valuable insights into the molecular composition of the sample. The prominent peak observed at 3427.51 cm^−1^ signifies O-H stretching vibrations [39], likely arising from hydroxyl groups present in both ascorbic acid and potentially residual water. Additionally, the peaks detected at 2924.09 cm^−1^ and 2854.65 cm^−1^ correspond to C-H stretching vibrations, suggesting the presence of aliphatic compounds, potentially originating from the organic ligands in the nanoparticles and ascorbic acid [40,41]. Additionally, the peak at 1610.56 cm^−1^ indicates C=C stretching vibrations, which are characteristic of aromatic compounds, and are likely associated with ascorbic acid or the organic ligands in the nanocomposite [42]. These findings collectively underscore the presence of organic constituents within the sample. Furthermore, the peaks at 1460.11 cm^−1^, 1431.18 cm^−1^, and 1363.67 cm^−1^ are indicative of C-H bending vibrations, commonly found in alkanes, potentially originating from the organic constituents of the nanocomposite [43]. The presence of peaks at 1078.21 cm^−1^ and 871.82 cm^−1^, attributed to C-O stretching vibrations, typical in alcohols, ethers, and esters, further supports the presence of ascorbic acid. Finally, the peaks observed at 823.60 cm^−1^, 727.16 cm^−1^, 630.72 cm^−1^, 547.78 cm^−1^, and 459.06 cm^−1^ are likely associated with metal-oxygen (Cu=O) in the Cu_2_O/AA NPs [44].

Upon comparing the FTIR spectra of pure Cu_2_O NPs with those of the Cu_2_O/AA NPs samples, a notable decrease in O-H peak intensity is observed in pure Cu_2_O NPs compared with Cu_2_O/AA NPs, suggesting an alteration in the composition of or interactions between functional groups upon the incorporation of ascorbic acid.

### 3.3. XRD Pattern Analysis

**Figure 3:**
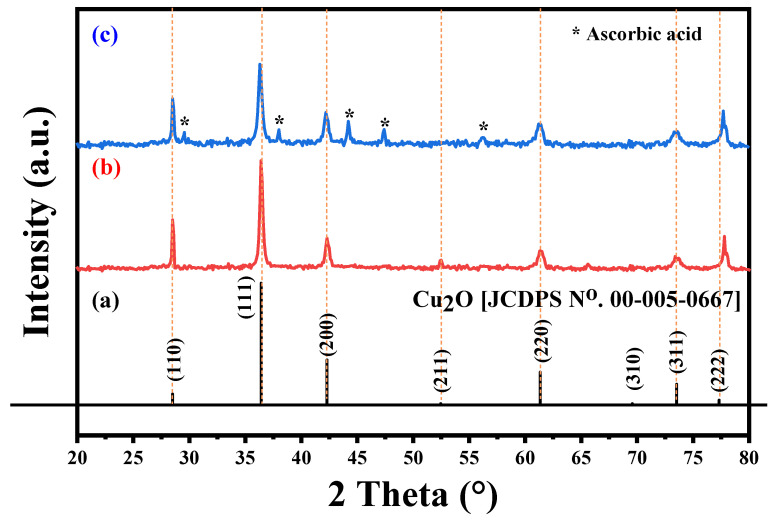
XRD patterns of (**a**) Cu_2_O standard XRD pattern [JCDPS no. 00-005-0667], (**b**) pure Cu_2_O, and (**c**) Cu_2_O/AA NPs.

The X-ray diffraction patterns of Cu_2_O NPs and Cu_2_O/AA NPs are illustrated in Figure 3a. The pure Cu_2_O sample (Figure 3b) exhibited sharp peaks located at two-theta positions at 29.555°, 36.419°, 42.298°, 52.455°, 61.345°, 69.571°, 73.528°, and 77.326° correspond to crystal planes 110, 111, 200, 211, 220, 310, 311, and 222 of Cu_2_O, respectively, which is supported by JCPDS card number [00-005-0667] [45], confirming the cubic crystal system of the Cu_2_O phase. Upon modification of the Cu_2_O NPs with ascorbic acid to form Cu_2_O/AA NPs (Figure 3c), the XRD pattern displayed the same peaks as pure Cu_2_O NPs, with additional peaks at 29.58°, 38°, 44.16°, 47.33°, and 56.2°. The additional peaks corresponding to the presence of the AA phase indicate the successful coating of Cu_2_O NPs with ascorbic acid [46], without significant alteration of the Cu_2_O crystal structure.

By employing Scherrer’s equation, which relates crystallite size (D) to various parameters including the form factor (k), wavelength (λ), Full Width at Half Maximum (FWHM) denoted by β, and diffraction angle (θ) [47], the crystallite sizes were calculated as 22.04 nm and 28.08 nm for Cu_2_O/AA NPs and pure Cu_2_O NPs, respectively. This decrease in crystallite size could be attributed to the presence of ascorbic acid on the surface of Cu_2_O NPs, which might inhibit the growth of crystalline domains or induce some level of structural disorder.

### 3.4. SEM Analysis

**Figure 4:**
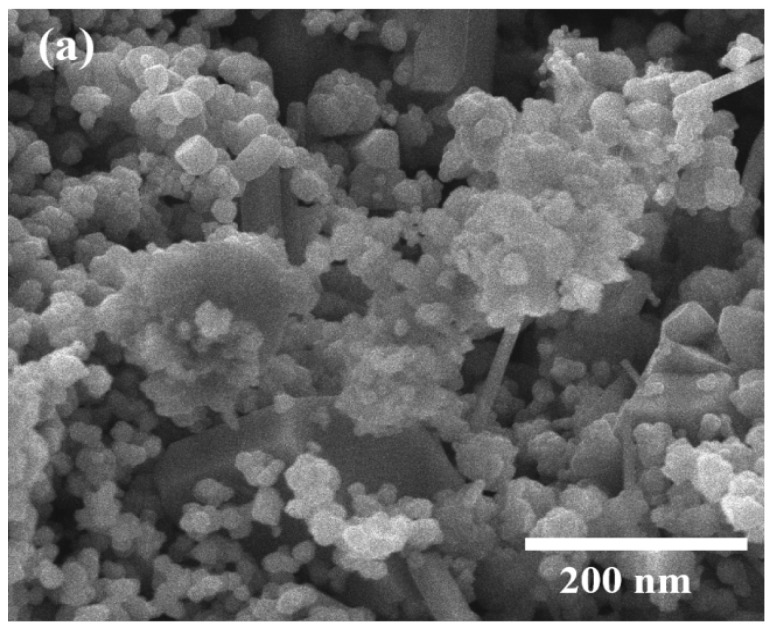

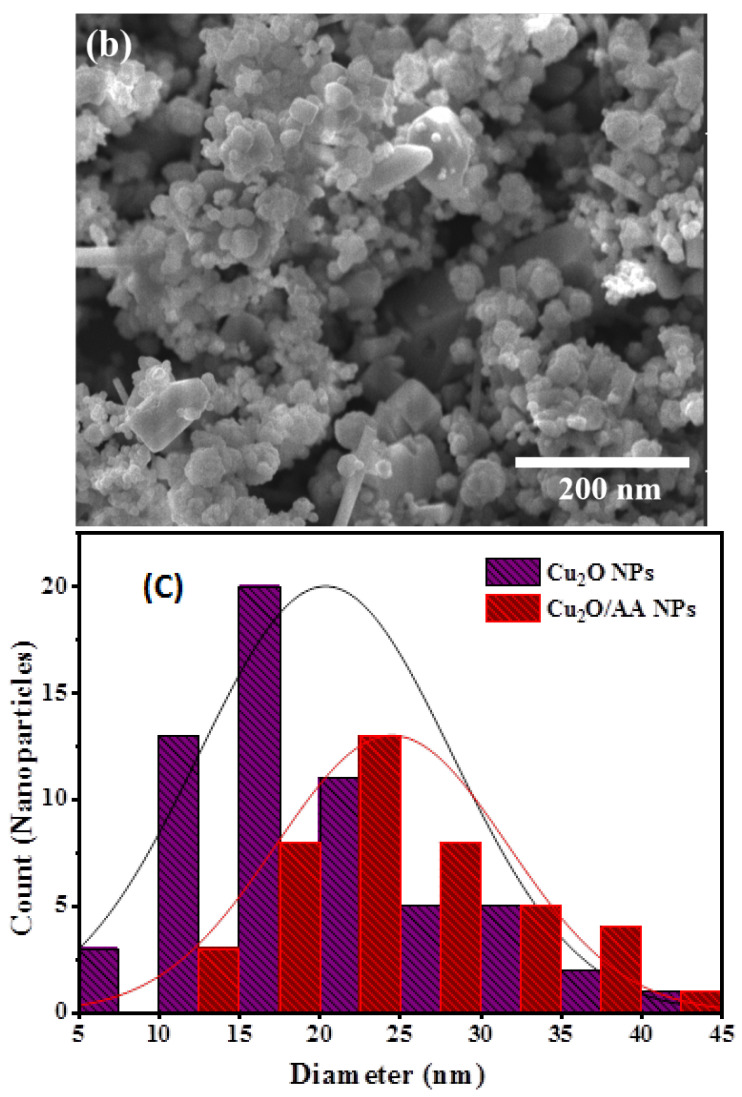
SEM analysis of (**a**) Cu_2_O NPs, (**b**) Cu_2_O/AA NPs; (**c**) particle size diameter of Cu_2_O NPs and Cu_2_O/AA NPs.

The SEM analysis revealed distinct morphological characteristics of both Cu_2_O NPs and Cu_2_O/AA NPs (Figure 4a,b). The SEM images provided a detailed view of the particle morphology, showcasing Cu_2_O NPs with a uniform spherical shape and an average size distribution of approximately 20 nm (Figure 4c). This uniformity in shape and size suggests a controlled synthesis process, indicative of the stability and reproducibility of the fabrication method. In contrast, Cu_2_O/AA NPs exhibited a more varied morphology, featuring a combination of spherical and irregular shapes. The average size distribution of these particles was slightly larger, around 25 nm, compared to pure Cu_2_O NPs. Moreover, the SEM images revealed densely packed particles on the surface of the Cu_2_O/AA NPs with minimal interparticle space, suggesting a high degree of packing and aggregation. This observation may arise from the interaction between Cu_2_O NPs and the ascorbic acid modifier, potentially leading to enhanced stability and surface coverage.

### 3.5. Zeta Potential Measurements

**Figure 5:**
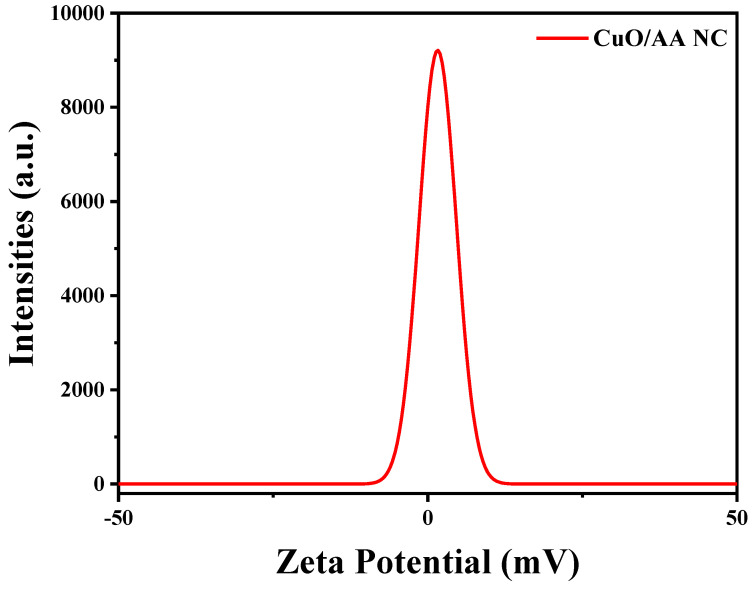
Zeta potential of the Cu_2_O/AA NPs.

The zeta potential of the Cu_2_O/AA NPs was determined to be approximately −1.76 mV, indicating that there was a negative surface charge on the modified nanoparticles (Figure 5). This negative value suggests stability in the deionized water dispersion medium used for the measurement. Although the magnitude of −1.76 mV is relatively low compared to highly stable colloidal systems (typically >+30 mV or <−30 mV), it still signifies significant electrostatic repulsion among the particles, playing a crucial role in preventing aggregation or agglomeration and enhancing colloidal stability. Consequently, the obtained zeta potential value implies that the Cu_2_O/AA NPs could be suitable for applications where surface charge and colloidal stability are critical factors, such as drug delivery systems, where nanoparticles need to remain dispersed and stable in biological fluids for effective delivery and transport [48]. Additionally, the stable dispersion of nanoparticles is essential in catalytic applications, where a high surface area and homogeneous distribution of the catalyst are desired for optimal performance. However, it is important to note that zeta potential is just one factor influencing colloidal stability, and other factors like particle size, shape, and surface chemistry also play important roles [49,50].

### 3.6. BET Analysis

**Figure 6:**
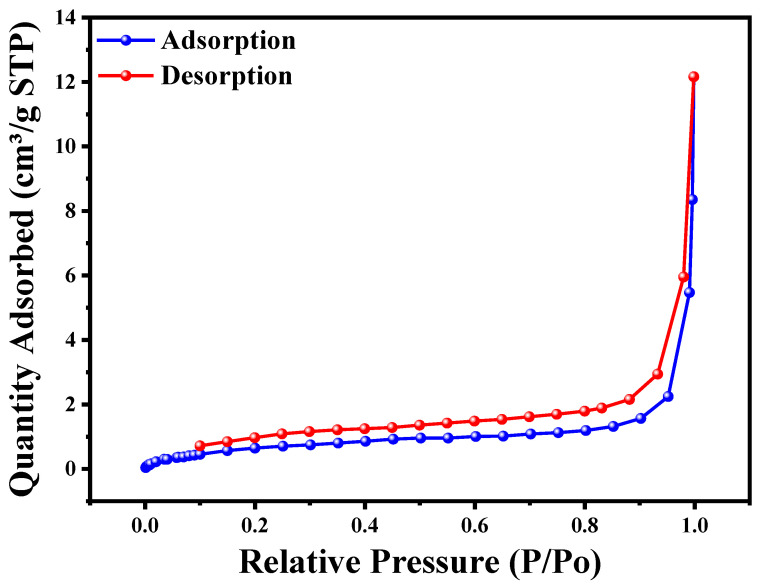
BET analysis for Cu_2_O/AA NC by using N_2_ adsorption-desorption isotherms.

The nitrogen adsorption–desorption isotherms shown in Figure 6 and the data in Table 1 provide insights into the porous structure and surface area of the Cu_2_O/AA NPs material. The BET surface area of 2.72 m^2^/g indicates a relatively low surface area, but the material is classified as mesoporous with an average pore diameter of 1.47 nm (BJH adsorption) or 1.07 nm (BJH desorption) in the range of 1–3 nm. Pore volumes of 0.0034 cm^3^/g (adsorption) or 0.0045 cm^3^/g (desorption) are relatively low, and the average particle size of 22.02 nm suggests the material consists of nanoparticles. The surface analysis of Cu_2_O/AA NPs using N_2_ adsorption–desorption isotherms revealed their mesoporous nature and promising catalytic behavior. The Type IV isotherm with a hysteresis loop confirmed the ordered mesoporous framework texture. While the specific surface area value is not exceptionally high, the presence of mesopores and the nanoparticle size may contribute to the reported “enormous photocatalytic activity” of the Cu_2_O/AA NPs material. The noticeable improvement in the specific surface area value and the superior N_2_ adsorption behavior of the Cu_2_O/AA NPs suggest their potential for high photocatalytic activity. The mesoporous structure, high surface area, and favorable pore characteristics of these nanoparticles are advantageous for catalytic applications. Overall, the results suggest that the Cu_2_O/AA NPs material possesses a mesoporous structure with a relatively low surface area and pore volume but a favorable nanoparticle size, which may contribute to its reported photocatalytic activity [51].

### 3.7. Photocatalytic Degradation of Brilliant Cresyl Blue

**Figure 7:**
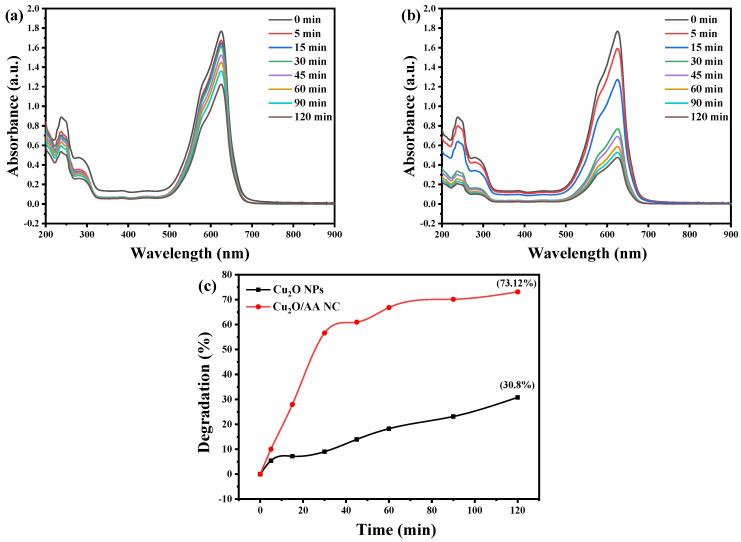
Photocatalytic behavior of BCB using (**a**) Cu_2_O NPs and (**b**) Cu_2_O/AA NPs at different irradiation times under UV-Vis irradiation. (**c**) Rate of degradation under sunlight exposure in the presence of Cu_2_O NPs and Cu_2_O/AA NPs.

The absorption spectra presented in Figure 7a–c provide a visual representation of the photocatalytic degradation of BCB dye by pure Cu_2_O NPs and Cu_2_O/AA NPs under solar light irradiation. The distinct absorption peaks observed at around 625 nm for BCB serve as a spectroscopic fingerprint, enabling the monitoring of the degradation process. As the photocatalytic reaction progresses, these characteristic peaks exhibit a gradual decrease in intensity, indicating the progressive breakdown of the dye molecules over an extended period.

The absorption spectra reveal a notable reduction in peak intensity for BCB dye, manifesting as a visible fading of its vibrant color over time. Quantitative analysis reveals that the photocatalytic activity towards BCB degradation achieves coefficients of 30.8% and 73.12% for Cu_2_O NPs and Cu_2_O/AA NPs, respectively, within a 120 min duration. Significantly, the Cu_2_O/AA NPs exhibit an improved decomposition efficiency over time, corroborating the findings of previous studies [52,53,54]. This outcome suggests the superior dye removal capability of the Cu_2_O/AA NPs, likely attributable to the synergistic effects arising from the composite structure and the presence of AA as a hole scavenger.

The gradual decrease in the absorption peak intensities indicates the progressive breakdown of the dye molecules, facilitated by the photocatalytic activity of the Cu_2_O NPs and Cu_2_O/AA NPs. The enhanced performance of the Cu_2_O/AA NPs can be attributed to several factors, including the composite structure, which may facilitate charge separation and prolong the lifetime of charge carriers, thereby improving the overall photocatalytic efficiency. Additionally, the presence of AA as a hole scavenger can potentially mitigate the recombination of photogenerated electron–hole pairs, further enhancing the photocatalytic activity.

The photocatalytic degradation of BCB dye using pure Cu_2_O NPs and Cu_2_O/AA NPs under solar light irradiation involves a series of photochemical reactions that ultimately lead to the breakdown of the organic dye molecules. When exposed to solar radiation, the semiconductor properties of Cu_2_O NPs and Cu_2_O/AA NPs initiate a cascade of events that drive the degradation process [51,55].

**Figure 8:**
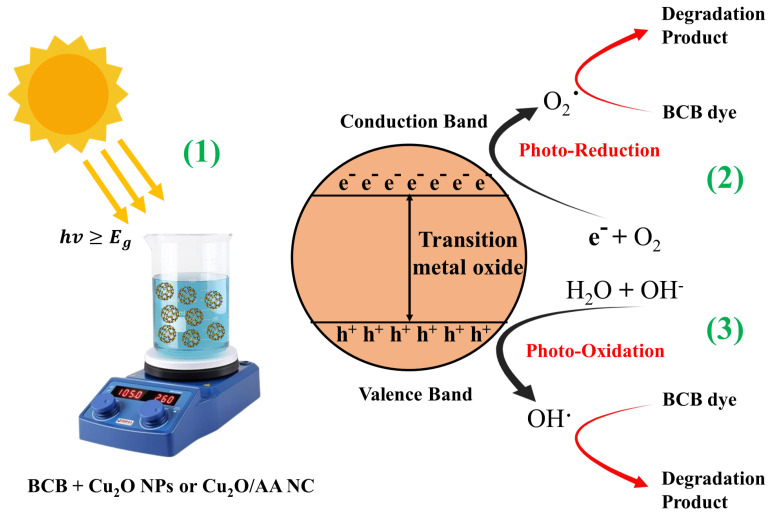
Photodegradation mechanism of BCB using Cu_2_O NPs and Cu_2_O/AA NPs.

Upon absorbing solar photons, electron–hole pairs are generated within the semiconductor materials. These photoinduced charge carriers migrate to the surface of the photocatalyst, where they participate in redox reactions with the adsorbed dye molecules. Specifically, electrons in the conduction band (CB) of Cu_2_O NPs and Cu_2_O/AA NPs can reduce the dye molecules, while holes in the valence band (VB) facilitate water oxidation, resulting in the formation of reactive oxygen species (ROS) such as hydroxyl radicals (${OH}^{\cdot }$) and superoxide radicals ($O_{2}^{\cdot -}$) (Figure 8).

These highly reactive ROS act as potent oxidizing agents, initiating an attack on the adsorbed BCB molecules and kickstarting the degradation process. This degradation involves breaking chemical bonds and cleaving chromophoric groups, leading to the transformation of complex dye molecules into smaller, less chromatic fragments. The presence of Cu_2_O NPs and Cu_2_O/AA NPs can significantly influence the overall efficiency of the photocatalytic procedure [37,56].

The key reactions involved in the photocatalytic degradation process can be summarized as follows:

1. Generation of electron–hole pairs: ${Cu}_{2}O/{Cu}_{2}O/AA\;NPs+hv\to {e^{-}}_{\left(CB\right)}+{h^{+}}_{\left(VB\right)}$;
2. Water oxidation and hydroxyl radical formation: ${h^{+}}_{\left(VB\right)}+H_{2}O\;\to \;H^{+}+{OH}^{\cdot }$;
3. Superoxide radical formation: ${e^{-}}_{\left(CB\right)\;}+O_{2}\to \;O_{2}^{\cdot -}$;
4. Additional hydroxyl radical formation: ${h^{+}}_{\left(VB\right)}+{OH}^{-}\to \;{OH}^{\cdot }$;
5. Degradation of *BCB* dye by ROS:
  $${OH}^{\cdot }+BCB\;\left(dye\right)\to \;{CO}_{2}+H_{2}O+Degradation\;product\;Dye+h^{+}\left(VB\right)\to oxidation\;products$$
6. Degradation of *BCB* dye by ROS:
  $$O_{2}^{\cdot -}++BCB\;\left(dye\right)\to \;{CO}_{2}+H_{2}O+Degradation\;product\;Dye+e^{-}\left(CB\right)\to reduction\;products$$

The photocatalytic degradation process involves a complex interplay between these photochemical reactions, ultimately leading to the breakdown of the organic *BCB* dye molecules. Here is a breakdown of the steps, going from left to right:

1. Light excitation (hv ≥ Eg): A light particle (hv) with energy greater than or equal to the bandgap (Eg) of the metal oxide excites an electron from the valence band to the conduction band.

2. Photo-oxidation: The positively charged hole (h^+^) in the valence band can directly oxidize pollutants or generate hydroxyl radicals (OH^·^) from water.

3. Photo-reduction: The excited electron (e⁻) in the conduction band can reduce molecular oxygen (O_2_) to superoxide radicals ($O_{2}^{\cdot -}$).

The Cu_2_O/AA NPs exhibit several distinct advantages over Cu_2_O NPs in the degradation of BCB dye: Firstly, the presence of the Cu_2_O component in the Cu_2_O/AA NPs enables the efficient utilization of visible light, which constitutes a significant portion of the solar spectrum, increasing the overall photocatalytic activity. Secondly, the heterojunction formed between Cu_2_O and AA facilitates efficient charge separation, reducing recombination and promoting the generation of ROS. Moreover, the incorporation of AA in the Cu_2_O/AA NPs promotes the formation of highly oxidizing ROS, which are essential for the effective degradation of organic pollutants like BCB dye, with AA not only acting as a capping agent but also contributing to the generation of these reactive species, thereby enhancing the photocatalytic performance. Additionally, the AA capping agent helps to stabilize the Cu_2_O/AA structure, preventing agglomeration and improving the reusability of the photocatalyst, a crucial factor for practical applications. Overall, the Cu_2_O/AA NPs represent a promising photocatalytic system for the degradation of BCB dye and other organic pollutants in wastewater treatment applications, offering several advantages over Cu_2_O NPs alone, including enhanced visible light absorption, improved charge separation, increased ROS generation, and better stability, making it an attractive candidate for efficient and sustainable wastewater remediation strategies.

Table 2 presents a comprehensive comparison of the photocatalytic degradation efficiency of various NPs, as well as Cu_2_O/AA, in the context of BCB degradation. Each entry in the table denotes the sample name, synthesis method, experimental conditions, catalysis dose, and degradation efficiency (% Deg) achieved within specified timeframes.

### 3.8. Recyclability and Stability

**Figure 9:**
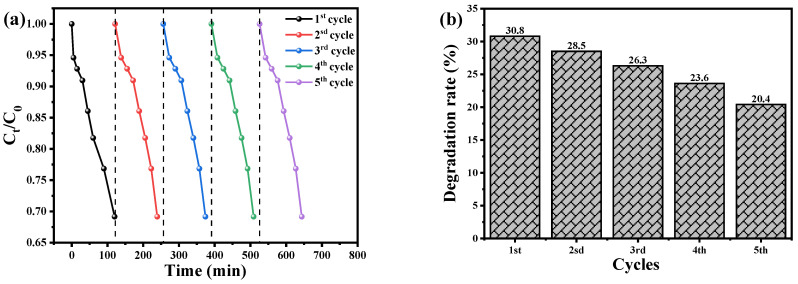

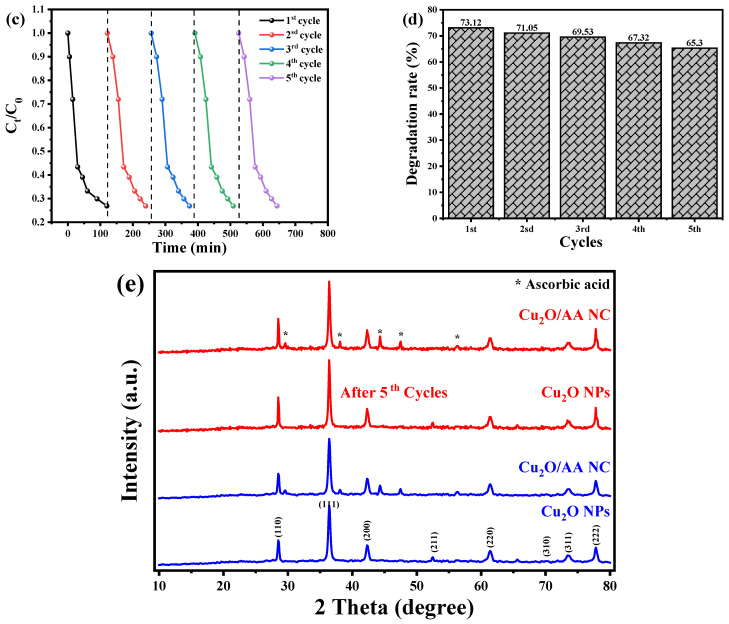
The recyclability of Cu_2_O NPs and Cu_2_O/AA NPs photocatalysts for degradation of BCB dye. (**a**) Cu_2_O NPs, (**c**) Cu_2_O/AA NPs, dotted line detain the start point of the Cycle and the next cycle and (**b**,**d**) reusability (degradation) efficiency vs. number of cycles in the photodegradation of BCB dye by Cu_2_O NPs and Cu_2_O/AA NPs, respectively. (**e**) XRD analysis of Cu_2_O NPs and Cu_2_O/ASA NPs of pure and reused.

The recyclability and reusability of photocatalysts are crucial factors in assessing their feasibility for practical applications in water remediation. The authors conducted a series of experiments to evaluate the reusability of CuO nanoparticles (NPs) and Cu_2_O/AA NPs as photocatalysts for the degradation of BCB dye. The experimental procedure involved drying and reusing the photocatalysts for five consecutive cycles under identical conditions as the initial cycle. The results, depicted in Figure 9a,c, demonstrate that both the Cu_2_O NPs and Cu_2_O/AA NPs exhibited remarkable initial effectiveness and reusability in photodegrading BCB dye. However, a slight decrease in the decomposition efficiency was observed over the course of the five cycles. Specifically, the efficiency of the Cu_2_O NPs declined from 30.8% to 20.4%, while that of the Cu_2_O/AA NPs decreased from 73.12% to 65.3% (Figure 9b,d). This decline can be attributed to the inevitable loss of photocatalyst material during the recycling process, which may occur during washing, centrifugation, or due to the adsorption of intermediate species generated during the photocatalysis process [60,61]. Interestingly, the XRD data presented in Figure 9e revealed that the essential diffraction peaks of Cu_2_O NPs and Cu_2_O/AA NPs remained intact both before and after the photodegradation process, throughout the five consecutive photocatalytic cycles. This observation suggests that the catalytic material did not undergo significant structural alterations, highlighting the stability and suitability of these photocatalysts for reuse in water remediation applications.

The observed decline in decomposition efficiency over successive cycles could be attributed to the depletion of active sites due to catalyst agglomeration or leaching during the recycling process. Optimized techniques for catalyst recovery and regeneration might potentially mitigate these losses, thereby enhancing the overall efficiency. The consistent X-ray diffraction patterns suggest that the Cu_2_O and Cu_2_O/AA photocatalysts retain their crystalline integrity even after multiple cycles. This structural stability is a promising indicator of their long-term durability in water treatment applications. Nevertheless, further investigations are warranted to delineate the impact of prolonged usage on the photocatalytic activity and devise strategies to sustain optimal performance. Furthermore, evaluating the photocatalysts under diverse environmental conditions (e.g., pH, light intensity) and with a wide range of pollutant species would provide invaluable insights into their applicability in real-world wastewater remediation scenarios.

## 4. Conclusions

This study offers a comprehensive investigation into the synthesis, characterization, and application of Cu_2_O NPs and their ascorbic acid-modified derivatives for the efficient photocatalytic degradation of BCB dye. By employing a range of analytical techniques, including UV-Vis spectroscopy, FTIR, XRD, zeta potential measurements, and BET analysis, we have gained significant insights into the structural, optical, morphological, and surface properties of both Cu_2_O and Cu_2_O/AA NPs. Our findings reveal their remarkable efficiency in BCB dye degradation, with Cu_2_O/AA NPs demonstrating a degradation coefficient of 73.12% within 120 min, surpassing pure Cu_2_O NPs which achieved 30.8% degradation under similar conditions. This enhanced performance can be attributed to improved charge separation, increased ROS generation, and superior stability conferred by the presence of ascorbic acid. Furthermore, recyclability studies showcased promising reusability of the photocatalysts, albeit with slight efficiency declines over successive cycles. Cu_2_O/AA NPs exhibited sustained catalytic activity, retaining structural integrity throughout five consecutive cycles of BCB dye degradation, as evidenced by XRD analysis. In light of these findings, Cu_2_O/AA NPs emerge as promising candidates for effective and sustainable wastewater remediation strategies. Their superior photocatalytic performance, coupled with favorable recyclability and stability characteristics, underscores their potential applicability in addressing water pollution challenges. Future research endeavors could explore optimization strategies to further enhance catalytic efficiency and elucidate the broader applicability of these nanocomposites in diverse environmental remediation scenarios.

## Figures and Tables

**Table 1:** BET surface area and porosity of Cu_2_O/AA NC.

Samples		Cu_2_O/AA NC
Surface Area	BET Surface Area	2.72 m^2^/g
	Langmuir Surface Area	10.75 m^2^/g
	t-Plot external surface area	4.036 m^2^/g
Pore Volume	Single point adsorption total pore volume of pores less than 40.3122 nm diameter at p/p° = 0.95	0.0034 cm^3^/g
	Single point desorption total pore volume of pores less than 40.3122 nm diameter at p/p° = 0.95	0.0045 cm^3^/g
Pore Size	BJH Adsorption average pore diameter (4 V/A)	1.47 nm
	BJH Desorption average pore diameter (4 V/A)	1.07 nm
Nanoparticle Size	Average Particle Size	22.02 nm

**Table 2:** Comparative analysis of BCB degradation via photocatalysis using various NPs.

Sample Name	Synthesis Method	Dye	Experimental Conditions	Catalysis Dose g/L	Deg Efficiency /Time	Ref.
g-C_3_N_4_/ZnO	Co-precipitation method	BCB	V = 50 mL, 25 ppm, pH = 10 Visible Light (250 w) Dark Room 30 min	0.5	99.51%/1 h	[5]
Co_3_O_4_/Fe_2_O_3_	-	BCB	V = 100 mL, 38.6 ppm, pH = 10 Sun-light	1	97% 3 h	[57]
ZnO/CuO	Precipitation method	BCB	V = 100 mL, 15 ppm, Visible Light (250 w) Dark Room 30 min	0.15	97.30% 1.66 h	[53]
ZnO	Cold Plasma GAD	BCB	V = 25 mL, 7 ppm, T° = 20 °C, pH = 7 UV Light (λ = 365 nm—30 W) Dark Room 30 min	1	92.92% /2.5 h	[58]
TiO_2_	Commercial	BCB	V = 100 mL, 3 ppm, UV Light (200 W) Dark Room 20 min	2.6	74% /2 h	[59]
Al_2_O_3_	Commercial	BCB	V = 100 mL, 50 ppm, pH = 10 (8.44 mW/cm^2^ light intensity) Dark Room 30 min	1.7	92.87%/1 h Al_2_O_3_ + UV + 10 cm^3^/min air bubble	[6]
Ag/ZnO	Turkevich	BCB	V = 100 mL, 100 ppm, pH = 7.5 UV Light (λ = 625 nm) Dark Room 30 min	0.1	91%/200 min	[6]
Cu_2_O/AA	Co-precipitation method	BCB	V = 200 mL, 50 ppm, pH = 7 UV Light (λ = 625 nm) Sun-light	1	73.12%/2 h	This work

## Data Availability

The original contributions presented in the study are included in the article, further inquiries can be directed to the corresponding author.
